# Flavonoid Enhances the Glyoxalase Pathway in Cerebellar Neurons to Retain Cellular Functions

**DOI:** 10.1038/s41598-017-05287-z

**Published:** 2017-07-11

**Authors:** Joel Frandsen, Prabagaran Narayanasamy

**Affiliations:** Department of Pathology and Microbiology, College of Medicine, University of Nebraska Medical Center, Omaha, NE 68198-5900 USA

## Abstract

Oxidative stress is damaging to cells and contributes to aging and neurodegenerative disease. This state is mediated by production of imbalanced molecules, and reactive dicarbonyl compounds - mainly methylglyoxal. The glyoxalase pathway is an antioxidant defense system utilized to detoxify methylglyoxal and neutralize free radicals. Pathway dysfunction leads to overproduction and accumulation of toxic, prooxidant compounds. We hypothesize flavonoid treatment as a means to enhance the glyoxalase pathway’s ability to detoxify in neurons. This study found that flavonoid treatment in methylglyoxal treated cerebellar neurons increased the functioning of glyoxalase pathway by enhancing expression of glyoxalase-1 and glyoxalase-2 proteins, decreased cell death and increased cellular viability. Flavonoids also significantly contributed in the retention of synaptic functions (VGLUT1 and GAD65) in cerebellar neurons. In addition, flavonoids were found to be involved in pAkt - NF-κB signaling pathway through a reduction in phosphorylation of Akt. The data here show flavonoid compounds have the potential to protect the brain from aging and neurodegenerative disease.

## Introduction

The brain contains oxidizable substrates, low antioxidant activity, and a high rate of metabolism, making neural cells and tissue vulnerable to oxidative stress (OS) and aging^1–4^. OS is a state of stress placed on organism resulting from disequilibrium between production of prooxidant molecules and the ability to neutralize the reactive molecules^4–6^. The glyoxalase pathway is an antioxidant system crucial in defense against reactive dicarbonyl compounds like methylglyoxal (MG)^7^. Aging is an organism’s progressive decline in physiological and metabolic functions characterized by chronic, low-levels of inflammation^8^. Aging related inflammation is a perpetual cycle involving MG mediated production of ROS, immune activation, and release of signaling molecules^8, 9^. Elevated levels of OS and MG are found in accelerated aging and neurodegenerative diseases including Alzheimer’s, Parkinson’s, and autism spectrum disorder^10–12^.

**Figure 1:**
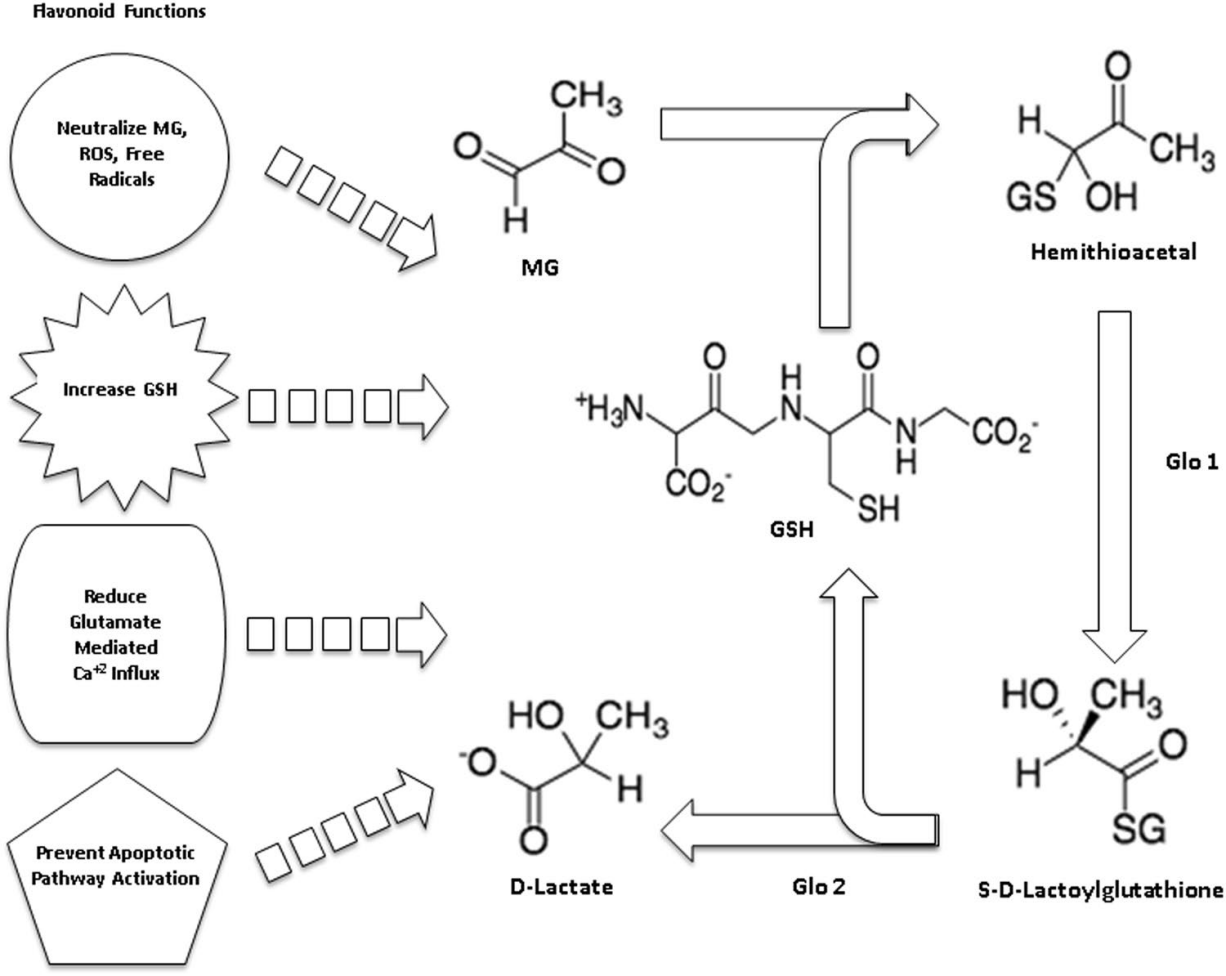
Scheme of flavonoid function in glyoxalase pathway. GSH reacts with MG to form a hemithioacetal. Glo-1 catalyzes the hemithioacetal into S-D-lactoylglutathione. Glo-2 catalyzes the final reaction, converting it into D-Lactate. Oxidized glutathione is used and recycled to its reduced form.

Detoxification of MG into D-lactate occurs through two sequential reactions^13^. D-Lactate can then be converted into pyruvate, the main substrate used to generate ATP through aerobic respiration^14^. The glyoxalase pathway is found in all bodily cells, and its function is twofold; it is an antioxidant defense system, and produces an alternate energy source for the cell^8^. Reduced glutathione (GSH) reacts with MG to form a hemithioacetal, allowing glyoxalase 1 (glo-1) to catalyze its conversion to an intermediate compound, S-D-lactyl glutathione^3^. Glyoxalase 2 (glo-2) catalyzes this molecule into D-lactate, fully completing the detoxification of MG while regenerating GSH in the process (Fig. 1)^11^.

Flavonoids are a class of polyphenol molecules conferred antioxidant ability through hydrophobicity and molecular structure, including multiple phenol rings, presence of unsaturated bonds, and a free hydroxyl group^15, 16^. They are able to directly scavenge free radicals, increase GSH concentration in cells, reducing glutamate mediated Ca^+2^ influx, and prevent activation of proapoptotic signaling pathways. The nuclear factor kappa-light-chain-enhancer of activated B cells (NF-κB) signaling pathway has broad control over cellular proliferation and apoptosis (Fig. 1)^17^.

Flavonoid antioxidants have the ability to protect neurons from MG cytotoxicity^18, 19^. Primary neurons treated with MG and flavonoids have higher levels of glo 1 and D-lactate compared to MG treated cells alone. Flavonoid treatment has different effects on different cell types. For example, quercetin treatment is highly toxic to cancer cells, but has few side effects on normal cells^20^. Flavonoid treatment on cancer cell lines exhibits a decrease in levels of glyoxlase I^21^. MG treatment at varying concentrations (50–200 μM) can inhibit cell proliferation, and at higher concentrations (1 mM) it causes cytotoxicity in neurons^22^.

This study investigated effects of flavonoids catechin, morin, and quercetin on enhancement of the glyoxalase pathway. The three well-known flavonoid antioxidant compounds - catechin, morin, and quercetin – possess free radical scavenging ability and very low cellular toxicity^16^. The purpose of this study is to provide evidence of flavonoid compounds’ ability to increase glyoxalase pathway efficiency to prevent neurodegenerative disease and aging of neurons. Flavonoids are able to achieve this through multiple mechanisms including: overexpression of glo-1, glo-2, increasing GSH, reducing concentration of ROS and free radicals, and modulating proapoptotic signaling pathways.

Cancer cell lines have an overexpression of glo-1 and MG, due to cellular response to cancer cell adaptation. Glo-1 overexpression is associated with cancer cell survival and resistance. In cancer cell lines, flavonoids act as inhibitors of glo-1. The polyvalent activity of flavonoids activates different cellular pathways resulting in proliferation or death of cancer cells.

## Results and Discussion

The main target for the glyoxalase system is MG: a highly reactive, membrane-permeable byproduct of glycolysis and other metabolic functions. Advanced glycation end products (AGEs) are formed from the interaction of MG with other biomolecules^1, 12^. AGEs have altered structure and reduced function relative to their normal counterparts, and are common markers of inflammation and OS^6, 11^. ROS are normal metabolic byproducts involved in cellular defense and used as signaling molecules^5, 15^. Because of this, the oxidation state of cells is always slightly in favor of prooxidant molecules^5^. Antioxidants directly scavenge free radicals, decompose ROS, chelate metal ions, and inhibit oxidative enzymes^5^. Antioxidants function by preventing formation of ROS, scavenging/neutralizing free radicals before they attack biological molecules, and repairing damage and loss of molecular function^23, 24^.

**Figure 2:**
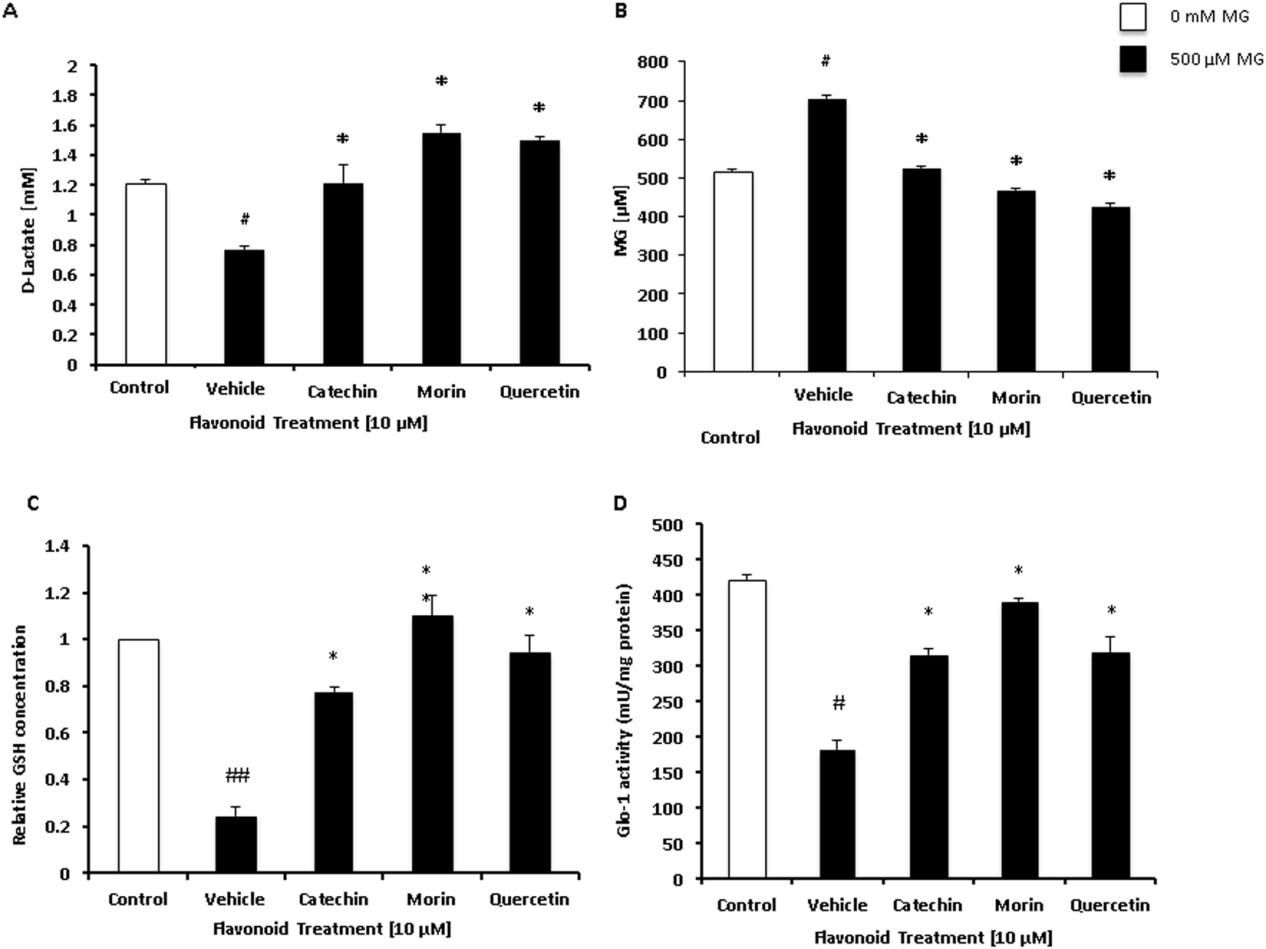
(**A**) Analysis of D-lactate release into extracellular space from neurons treated with MG (500 μM) and flavonoids (10 µM). Control = no MG, Vehicle = only MG. Results are means +/− SEM of multiple independent experiments performed in triplicate. ^#^P < 0.05 (vs Control), *P < 0.05 (vs Vehicle). (**B**) Concentration of MG (µM/mL) in extracellular space was determined in cerebellar neurons treated with MG (500 μM) and flavonoids (10 µM). Values are means +/− standard error of samples performed in triplicate from several independent experiments. ^#^P < 0.05 (vs Control), *P < 0.05 (vs Vehicle). (**C**) Concentration of GSH in cellular lysates was determined in cerebellar neurons treated with MG (500 µM) and flavonoids (10 µM). Values are means +/− standard error of samples performed in triplicate from several independent experiments. ^##^P < 0.01 (vs Control), *P < 0.05 (vs Vehicle), **P < 0.01 (vs Vehicle). (**D**) Glyoxalase 1 activity was determined in cerebellar neurons treated with MG (500 µM) and flavonoids (10 µM). Values are means +/− standard error of samples performed in triplicate from several independent experiments. ^#^P < 0.05 (vs Control), *P < 0.05 (vs Vehicle).

The accumulation of MG in cells is toxic and irreversibly modifies the proper structure of macromolecules. The glyoxalase system converts reactive MG into D-lactate, preventing OS mediated damage to the cell. Hence MG is used to induce the neurons for aging. The concentration of MG treatment was determined through D-lactate assay with varying concentrations of MG and observed 0.5 mM as optimal concentration with no cytotoxicity [Figure S1–S3]. The concentration of D-lactate in the extracellular space provides a means to measure the detoxification of MG by the glyoxalase system. D-lactate concentration was determined by spectrophotometrically measuring the conversion of NAD to NADH. For protecting from aging, cerebellar neuron cultures were incubated with MG (500 μM) and a 10 μM flavonoid (in dose response study 5 μM flavonoid did not show difference and 10 μM flavonoid showed significant difference) for 24 hours. Catechin, morin, and quercetin treated cerebellar neurons had a significantly higher concentration of D-lactate than the MG treated control. The media from MG treated cells contained 0.7 mM of D-lactate, while the flavonoid treated groups had D-lactate concentrations in excess of 1 mM. The increase in D-lactate concentration in the presence of flavonoid indicates that the glyoxalase pathway is enhanced and MG has been detoxified in high amount (Fig. 2A).

MG is cytotoxic, propagates the production of ROS and inflammatory molecules, and increases the expression of pro-apoptotic gene products^25^. Cerebellar neuron cultures were incubated with MG (500 μM) and 10 μM flavonoid (in dose response study 5 μM flavonoid did not show much difference and 10 μM flavonoid showed significant difference) for 24 hours. The reaction of MG with 2,4-DNPH produces dinitrophenylhydrazone, which was measured spectrophotometrically to determine the MG content. Catechin, morin, and quercetin were able to significantly decrease the concentration of MG compared to the control. Flavonoid treatment was able to attenuate the concentration of MG to levels of the non-treated control (Fig. 2B). This result matches when co-related with increase in lactate concentration (Fig. 2A).

GSH is a protein utilized for antioxidant defense. In the glyoxalase pathway, it catalyzes the first step in the conversion of MG to D-lactate. High concentration of MG depletes the intracellular concentration of GSH, preventing efficient functioning of the glyoxalase pathway. In cerebellar neurons MG treatment drastically decreased the concentration of GSH. Flavonoid treatment was able to attenuate the MG toxicity, leading to significantly higher GSH concentrations. Morin and quercetin were able to increase GSH levels to that of the untreated control (Fig. 2C).

The glyoxalase activity assay evaluated the capacity of the cerebellar neurons to detoxify MG. Glyoxalase activity measures the mU/mg protein needed to catalyze the conversion of the GSH-MG hemithioacetal into S-Lactoylglutathione. MG treatment of 500 µM significantly reduced the activity of glo-1 in cerebellar neurons. Flavonoid treatment decreased the glo-1 activity inhibition caused by MG. Morin was found to significantly increase glo-1 activity compared to the MG treated vehicle. (Fig. 2D).

MG is cytotoxic, and its accumulation can induce neuronal death by promoting production of pro-apoptotic molecules in aging neurons. MG causes the activation and subsequent cleavage of caspase-3, which is a regulator of apoptosis and triggers cell death through a mitochondrial mediated pathway. Cerebellar neuron cultures underwent a 24 hour treatment with MG and flavonoids, and were fixed for immunocytochemistry. Cells were conjugated with antibodies directed towards cleaved caspase-3 (Cas-3) and neuronal specific nuclear protein (NeuN) to determine apoptosis and survival, respectively. Relative fluorescence was determined through confocal microscopy imaging. MG treatment significantly increased the amount of Cas-3 positive cells, and decreased the amount of NeuN positive cells compared to the vehicle.

**Figure 3:**
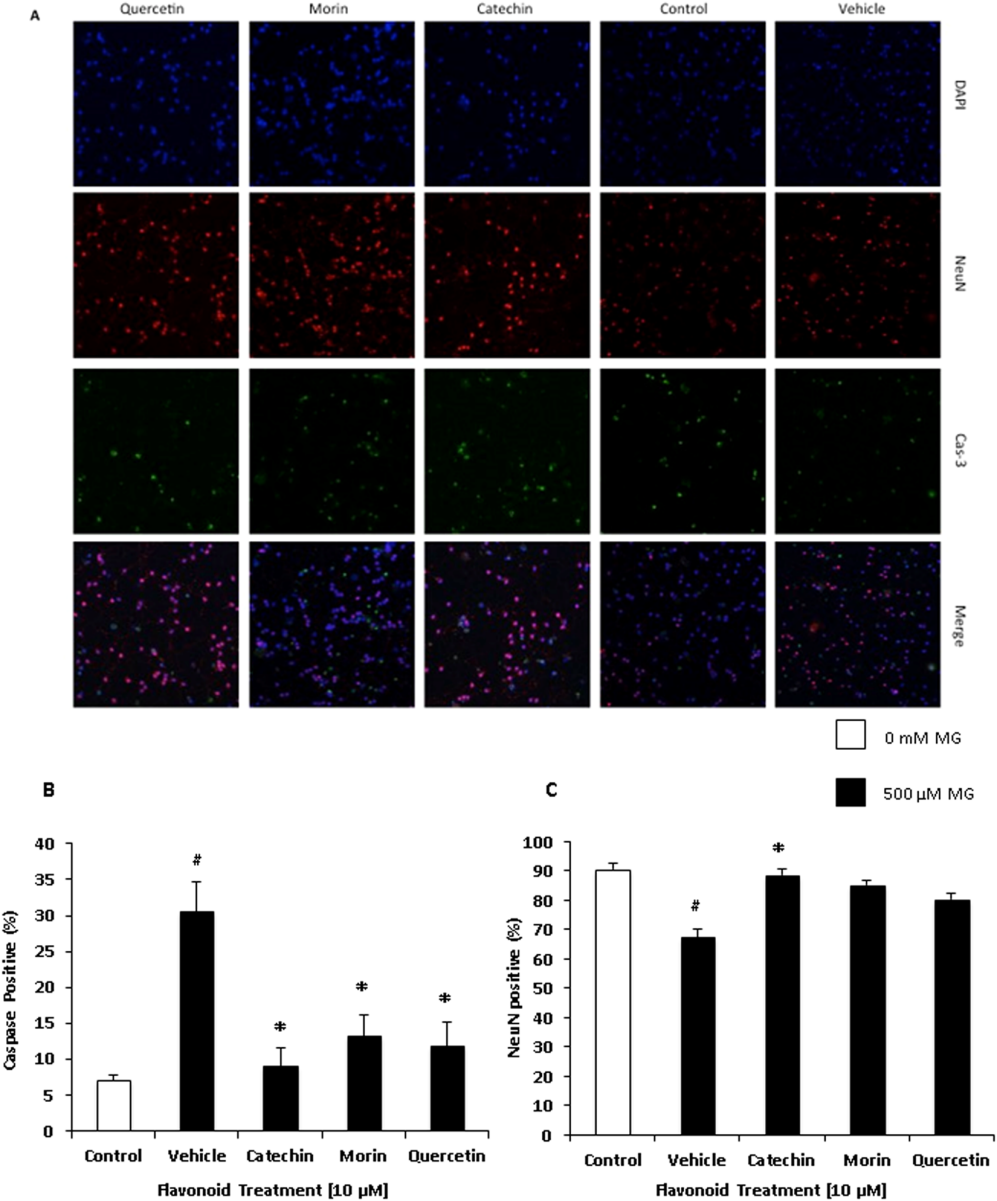
Effects of flavonoid treatment on cell viability and apoptosis. Cerebellar neurons were treated with flavonoid (10 µM) and MG (500 μM). Data was determined *via* immunocytochemistry (**A**). Viability was assessed through detection of cleaved caspase-3 (Cas-3) (**B**) and neuronal specific nuclear protein (NeuN) (**C**). Control = no MG, Vehicle = only MG. Values are means +/− SEM of samples performed in triplicate from several independent experiments, n = 3–4. ^#^P < 0.05 (vs Control), *P < 0.05 (vs Vehicle). Images were quantified with ImageJ, and resulting data were analyzed using an ANOVA. Morin treatment significantly decreased the presence of apoptosis, and catechin and morin significantly increased cell viability.

A significant decrease in the amount of Cas-3 positive cells was found in the catechin, morin, and quercetin treated conditions (Fig. 3B). A significant increase in the amount of NeuN positive cells compared to the control was found in the catechin and morin treated conditions (Fig. 3C). These results suggest flavonoid treatments increase survivability of cerebellar neurons in the presence of cytotoxic MG.

**Figure 4:**
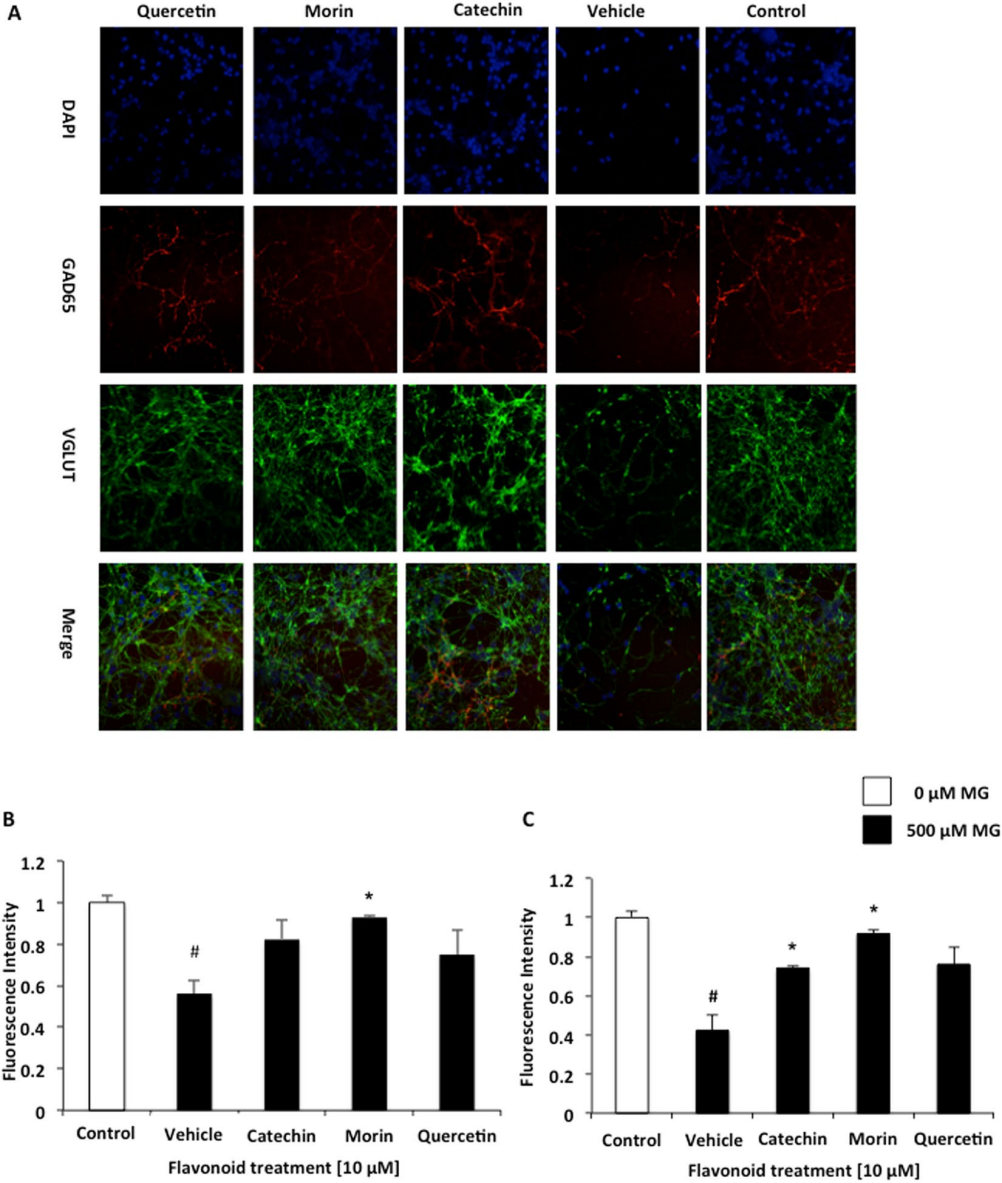
Effects of flavonoid treatment on excitatory and inhibitory synaptic functions. Functions were assessed through immunocytochemistry. Representative images showing DAPI, VGLUT1, and GAD65 in flavonoid (10 µM) and MG (500 μM) treated cerebellar neurons (**A**). Excitatory and inhibitory synapse function was assessed through detection of VGLUT1 (**B**) and GAD65 (**C**). Control = no MG, Vehicle = only MG. Values are means +/− SEM of samples performed in triplicate from several independent experiments, n = 3–4. ^#^P < 0.05 (vs Control), *P < 0.05 (vs Vehicle).

MG accumulation in cells leads to an increase in ROS, which can disrupt and compromise the structure and function of neural synapses in aging neurons. The accumulation of neurotransmitters in the synapses can alter signaling and lead to apoptosis. Accumulation of glutamate in the synaptic cleft leads to an increased influx of Ca^+2^, which can culminate in cell death. Excitatory and inhibitory synapse function was measured using antibodies directed towards vesicular glutamate transporters (VGLUT1) and glutamic acid decarboxylase (GAD65) respectively. Here, we assessed the number of excitatory and inhibitory synapses using synaptic markers VGLUT1 and GAD65 in the neurons on treatment with MG and drug like compounds. As expected on treatment with 0.5 mM MG, the VGLUT1 and GAD65 numbers reduced in cerebellar neurons. Flavonoid treated cells had a significantly higher concentration of VGLUT1 than the MG treated controls. Glutamic acid decarboxylase catalyzes the conversion of glutamate to GABA. Inhibitory synapse function was elucidated through cerebellar neurons conjugated with GAD65. Interestingly the VGLUT1 and GAD65 survived in presence of morin, showing the potential of morin to be a drug-like compound for neuronal growth. Flavonoid treated neurons had a significantly higher content of GAD65 activity than the MG treated control (Fig. 4).

**Figure 5:**
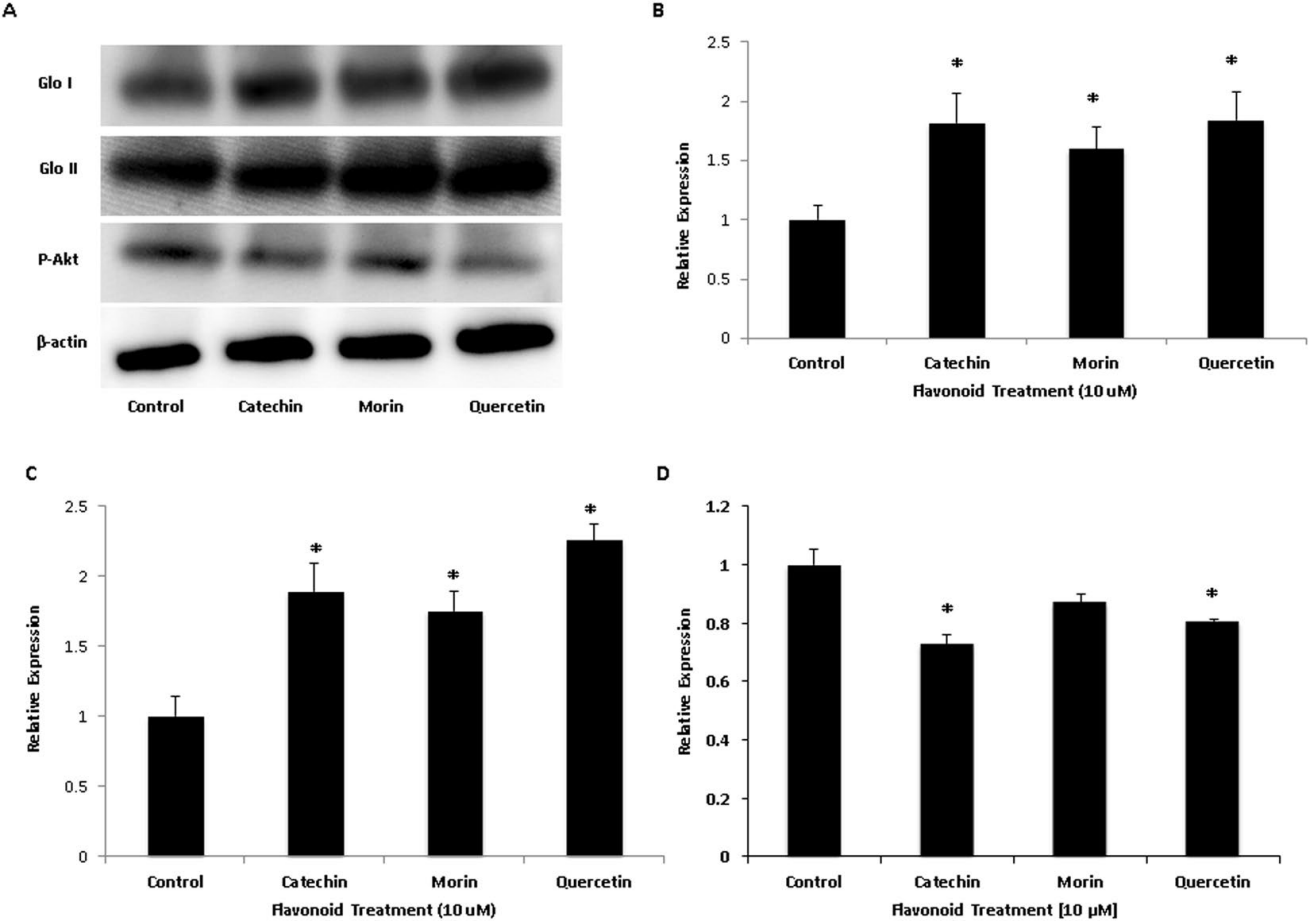
Effect of flavonoid treatment on expression of proteins glo-1 (**B**), glo-2 (**C**), and pAkt (**D**). Representative Western Blot images (**A**). Values are means +/− SEM of samples performed in triplicate from several independent experiments, n = 3–4. *P < 0.05 (vs Control). Expression of P-Akt in cerebellar neurons was assessed via Western Blotting. Cerebellar neurons were treated with MG (500 μM) and flavonoids (10 µM). Blots were conjugated with β-actin to ensure equal protein loading. Control = only MG. Values were determined using ImageJ. Resulting data were analyzed using an ANOVA. A significant decrease (*p < 0.05) in P-Akt was found in the catechin and quercetin treated groups (10 μM) compared to the MG treated control.

The efficiency of the glyoxalase system to detoxify MG is predicated upon the presence and activity of glo-1 and glo-2 proteins^26, 27^. Increased expression of the proteins allows for increased detoxification of MG. Cellular lysates from MG and flavonoid treated cerebellar neurons were used for a western blot to determine the relative expression of glo-1 and glo-2. Flavonoid treatment increased the expression of glo-1 in cerebellar neurons, leading to a significant increase in expression compared to the control (Fig. 5B). Glo-2 expression was also increased by flavonoid treatment, having a significant increase (*p < 0.05) compared to the control (Figs 5C and S5).

Flavonoids have the ability to reduce production of ROS, inflammatory cytokines, and prevent transcription and expression of apoptotic signaling cascades. NF-κB is composed of two subunits (p65/p50), whose activation by phosphorylation is necessary for nuclear translocation and transcription. ROS and MG activate IKKβ, allowing NF-κB to able to translocate to the nucleus and upregulate transcription of apoptotic proteins in aging neurons. The NF-κB signaling pathway can be activated through multiple mechanisms. The PI3K-Akt pathway is able to trigger activation through phosphorylation of subunits. Flavonoid treatment of cerebellar neurons reduced expression of p-Akt in the presence of MG (Fig. 5D).

Cerebellar neurons have been established as a model for OS and cytotoxicity, making it appropriate for studies on aging and neurodegeneration^28^. Aging has several distinct hallmarks, including increased production of ROS and inflammatory molecules, disruption of signaling networks, and deficits in endogenous antioxidant defense systems. Evidence has shown antioxidant enhancement of the glyoxalase system can negate and prevent the damage caused by MG mediated OS. Lowering the level of OS in the brain can reduce the severity and prevalence of aging and related disease.

This study investigated the effects of flavonoids on glyoxalase system function. We hypothesized that flavonoids would enhance the efficiency of the glyoxalase system in cerebellar neurons by multiple mechanisms: directly reducing concentration of free radicals, increasing concentration of GSH, overexpression of glo-1, and glo-2, decreasing apoptosis, and modulating proapoptotic signaling pathways. At a relatively low dose (10 μM), flavonoids proved to be effective in protecting cerebellar neurons from MG (500 μM) mediated damage. Flavonoids enhanced the glyoxalase system by increasing MG detoxification into D-lactate, and expression of constituent pathway proteins, and also reduced expression of apoptotic proteins.

Flavonoid treatment attenuated MG toxicity in cerebellar neurons, and lead to an increased concentration of D-lactate in the extracellular media. Flavonoid treatment was able to increase the efficiency of the glyoxalase system even under conditions of extreme cytotoxicity. In accordance with this, we observed a decreased concentration of MG in the media. Cerebellar neurons increased the rate of their endogenous antioxidant system through flavonoid supplementation. Reducing the concentration of MG will reduce the production of ROS and proinflammatory cytokines. It will also decrease the amount of oxidized molecules and AGEs, which could provoke a chronic immune response and directly lead to conditions of OS. Reducing the oxidative burden on the glyoxalase system allows its antioxidant functions to be more efficient and prevent the exponential rise of toxic molecules in the cellular milieu.

Flavonoids increased the expression of both glo-1 and glo-2 proteins in cerebellar neurons. In general, lowered expression of the constituent pathway proteins has been linked to premature aging, aging, and increased severity of neurodegenerative disease. Low levels of glyoxalase proteins also cause an increase in apoptosis, AGEs, and other oxidized molecules. Increased expression of glyoxalase proteins has been shown to lower formation of ROS and free radicals, and elevated levels corresponded to a decrease in concentration of alpha-oxoaldehydes. MG detoxification requires the presence of both glo-1 and glo-2. A decrease in either pathway protein would be rate limiting and prevent efficient pathway function. The accumulation of MG - caused by deficits in the pathway - gradually increases the rate of neurodegeneration in Alzheimer’s Disease and related dementia. The absence of glo-1 and glo-2 has a dramatic negative impact on neuronal viability, and the system’s efficiency depends on the presence and activity of its constituent proteins and cofactors. Aging and high OS can cause glo-1 and glo-2 to be under expressed to lower levels. Severity of neurodegenerative disease is influenced by the levels of OS. This study showed the effectiveness of flavonoid treatment against MG mediated toxicity.

High levels of OS are detrimental to the structure and function of neuronal synapses. MG induced toxicity can disrupt the synaptic morphology, effecting the uptake and release of neurotransmitters. A damaged receptor will not efficiently send or respond to the brain’s chemical messages. Accumulation of glutamate in the synapses is a trigger for a cellular influx of Ca^2+^, which initiates an apoptotic signaling cascade. Flavonoid treated cerebellar neurons were able to effectively retain the structure and function of both excitatory and inhibitory synapses. We found flavonoid treatment positively increased markers of glutamate and GABA receptor function in the presence of MG. These neurons were able to more efficiently convert glutamate to GABA, and clear glutamate from the synaptic cleft. Flavonoids also had a significant impact on cell viability by decreasing the activated form of caspase-3. Cleavage of this protein initiates mitochondrial mediated apoptosis, releasing other pro-apoptotic proteins and resulting in destruction of the mitochondria and death of the cell. This signaling cascade can also be initiated by increased concentration of ROS and inflammatory cytokines, production of both being mediated by MG.

NF-κB is initiated in response to cellular damage, inflammation, and cytokine release. The NF-κB heterodimer has two subunits (p65, p50) and is sequestered in the cytoplasm when bound to its inhibitory subunit, IκBα. Phosphorylation degrades the inhibitory subunit, and NF-κB is translocated to the nucleus for rapid initiation of apoptotic gene transcription. MG and ROS mediate inhibitory subunit phosphorylation, thus regulating the transcription of pro-apoptotic protein products. Decreasing MG and free radicals in the cell directly reduces the amount of molecules able to modulate and activate this signaling pathway.

Flavonoids also enhance the glyoxalase system by modulation of signaling pathways. The NF-κB signaling pathway can be initiated under situations of inflammation and infection. The pathway has a wide range of controls and functions, including apoptosis and proliferation. Activation of NF-κB in the cytoplasm causes its translocation to the nucleus, where it subsequently initiates transcription of apoptotic proteins. The inhibitory subunit binds to NF-κB and keeps it sequestered in the cytoplasm. Phosphorylation of IKKβ, leads to its ubiquitination and release of NF-κB. Phosphorylation occurs under a wide range of circumstances, but is influenced by the amount of free radicals and cytokines in the cellular environment. Flavonoids were found to modulate NF-κB signaling through a reduction in phosphorylation of Akt. NF-κB will degrade if left in the cytoplasm, and blocking its translocation will prevent gene transcription of pro apoptotic products. The amount of ROS in the cell influences immune response and inflammation. Flavonoids are able to directly scavenge free radical molecules, preventing their role in phosphorylation of NF-κB pathway constituents.

In primary cerebellar neurons, MG toxicity was significantly attenuated by flavonoid treatment and found to reduce oxidative stress. Flavonoids are able to protect against cellular damage by reducing methylglyoxal. It also increases the expression of glo-1 and glo-2. In addition flavonoids retain the VGLUT1 and GAD65 functions. These compounds are also involved in the pAkt - NF-κB signaling pathways. We found flavonoids offered protection against MG mediated OS in cerebellar neurons, preventing progression of neurodegenerative disorders. These metabolites offer an attractive solution against OS mediated damage to cells. They have very low cytotoxicity (Fig. S4), and function to increase the efficiency of the glyoxalase system. Proper function of the glyoxalase system decreases the amount of free radicals and damaging reactive compounds and retains neuronal function. Based on the results of this study, flavonoids compounds may prove to be an effective treatment for aging and neurodegenerative disease.

## Materials and Methods

### Care and use of animals

Animal studies were approved and performed in accordance with the UNMC Institutional Review Board (IRB) and Institutional Animal Care and Utilization Committee (IACUC). C57BL/6 mice breeding pairs were obtained from The Jackson Laboratory (Bar Harbor, ME).

### Chemicals and compounds

Morin was purchased from MP Biomedicals (Solon, OH). Quercetin dehydrate was purchased from Pfaltz & Bauer, (Waterbury, CT). Sodium D-lactate was purchased from Santa Cruz Biotechnology (Dallas, TX). Lactate dehydrogenase was purchased from US Biological (Salem, MA). Catechin Hydrate, Poly-D-lysine hydrobromide, β-nicotinamide adenine dinucleotide hydrate, methylglyoxal, and 2,4-Dinitrophenylhydrazine were purchased from Sigma Aldrich (St. Louis, MO). Unless otherwise noted, chemicals for this study were purchased from Thermo Fisher Scientific (Fair Lawn, NJ).

### Cerebellar neuron culture

Primary cultures of cerebellar neurons were prepared from P5 C57/BL6 mice. Six well culture plates (Falcon, Indianapolis, IN) were coated with poly-D-lysine and seeded with cerebellar neurons at a density of 1.5 × 10^6^ cells/well, and maintained with DMEM media supplemented with L-glutamine, pen-strep, 30% sucrose, B-27, and N-2. Ara-C was added 24 hours after seeding to produce a homogenous neuronal culture. Cells were grown to confluence for 6 days, with half media changes every 48 hours. The negative control received no treatment, the vehicle was treated with 500 μM MG. Antioxidant treatment groups were treated for 24 hours by adding 500 μM MG first and followed by 10 μM flavonoid.

### Western Blot Analysis

Cerebellar neuron cultures were washed with ice cold PBS three times and lysed using RIPA buffer (50 mM Tris, pH 7.4, 150 mM NaCl, 1% Triton X100, 1% Na deoxycholate 0.1% SDS, 1 mM EDTA) and a protease inhibitor cocktail (Thermo-Fisher). A BCA assay (Pierce) was used to ensure equal protein loading content. Samples were added to a loading buffer (LDS sample buffer 4X, B-mercaptoethanol, PBS) and denatured at 90 °C for 5 minutes. Proteins were resolved on a 4–20% tris-glycine gel (Bio-Rad) using SDS-PAGE, and transferred to a PVDF membrane (Immobilon). Membranes were blocked for 1 h in TBST and 5% fat free milk (BioRad), washed in TBST, and incubated overnight at 4 °C with antibodies directed against Glo-1 (Rabbit polyclonal, Santa Cruz), Glo-2 (Goat polyclonal, Santa Cruz), and p-Akt (Rabbit polyclonal, Cell signaling). Blots were washed and incubated with the appropriate HRP-conjugated secondary antibody (Santa Cruz). β-actin expression (Rabbit monoclonal, Cell Signaling) was assessed to ensure equal protein content.

### Determination of D-lactate concentration

Media samples from cerebellar cultures were collected prior to lysing the cells. D-Lactate released into the extracellular space following treatment with MG and antioxidant was measured spectrophotometrically. Culture media samples of 70 μL were loaded on a 96-well plate with 180 μL of glycine buffer (0.2 M glycine, 0.2 M semicarbazide, pH 10), containing 2 mg/mL NAD and 5 U/mL D-Lactate Dehydrogenase. Samples were incubated at room temperature for 2 hours. A spectrophotometer (340 nm excitation, 450 nm emission) was used to measure conversion of NAD to NADH. Absolute values were determined from a standard curve of D-Lactate concentrations^11, 29^.

### Determination of MG concentration

A 10 mM stock solution of 2,4-DNPH (Sigma-Aldrich, St. Louis, MO, USA) in 100% ethanol was used to create a working solution of 0.2 mM 2,4-DNPH with 12 mL HCl (36%) per 100 mL ethanol. A working solution of 1 mM MG (Sigma) was prepared from a stock solution. The reaction consisted of 950 µL 0.2 mM 2,4-DNPH with different volumes of 1 mM MG, filled to 1 mL with distilled water for the blank, or 50 mL of media from cerebellar neuron culture. The samples and blanks were heated in an Eppendorf Thermomixer at 42 °C for 45 mins and 600 rpm, and were incubated at room temperature for 5 minutes. Spectrophotometer measurements were performed at 432 nm, according to absorbance of MG-bis-2,4-DNPH-hydrazone for calculating concentration of MG.

### Glyoxalase I activity

Neurons were plated in 12 well plates at 5 × 10^5^, and grown to maturity at 6 days. Cells were treated with 500 μM MG and 10 µM antioxidant for 24 hours. Media was removed, and cells were rinsed with ice cold PBS. Cells were lysed with buffer containing: 10 mM HEPES (pH 7.0), 0.02% Triton X-100, and 100 µg/mL BSA. Samples were briefly sonicated and centrifuged at 16,000 g for 30 minutes at 4 °C. Cellular lysates were added to a 96 well plate at 50 µL per well. Reaction mix consisted of 60 mM sodium phosphate pH 6.6 containing 4 mM GSH and 4 mM MG. Two hundred µL reaction mix was added to the 96 well plate and incubated for 10 minutes at 37 °C, and 50 µL of sample lysate was added to the plate. S-lactoylglutathione synthesis was determined by measuring absorbance at 240 nm every 15 seconds for 5 minutes. Protein concentration was determined using a BCA protein assay reagent kit (Thermo Scientific)^11, 30^.

### Determination of GSH concentration

Total intracellular glutathione levels were determined spectrophotometrically as previously described^11, 29, 31^. Values were calculated from a standard curve of GSSG.

### Immunocytochemistry

Primary cerebellar neurons were harvested from P5 C57/BL6 mice. Neurons were plated in 8 well imaging plates at 300,000 cells/well. After growing for 6 days, cells were treated with 500 μM MG and 10 µM flavonoids for 24 hours. Media was removed and cells were fixed with 4% PFA/30% sucrose solution in PBS for 10 mins. Wells were washed with PBS, and 0.1% Triton 100x in PBS for 10 mins. Wells were again washed with PBS, and blocked with 5% BSA in PBS at room temperature for 1 hour. Primary antibodies for NeuN (mouse monoclonal) and cleaved caspase-3 (mouse monoclonal), and anti-VGLUT1 (Guinea Pig polyclonal) and GAD65 (rabbit monoclonal) were added in 1% BSA in PBS overnight. Wells were washed with PBS, and incubated with the appropriate IgG (H + L) conjugated secondary antibodies at room temperature for 1 hour. Wells were washed, and covered with DAPI stain for 1 minute. DAPI stained cells were rinsed off, wells were suctioned, and Prolong Gold Antifade (Thermo Fisher Scientific, MO) was added directly to each well. Plates were imaged on a confocal microscope (LSM 710 Zeiss Confocal Microscope) at 40x.

### Statistics

Statistics were performed using Excel (Microsoft) and SPSS (IBM Corporation). Data were expressed as the mean +/− SEM of multiple experiments performed in triplicate. Differences between means were statistically analyzed using a student’s t-test or one-way analysis of variance (ANOVA). Data with at least P < 0.05 was considered statistically significant.

## Electronic supplementary material

supporting information
