# Supplementary material for: Flavonoid Enhances the Glyoxalase Pathway in Cerebellar Neurons to Retain Cellular Functions
Source: Sci Rep. 2017 Jul 11;7:5126. doi: 10.1038/s41598-017-05287-z (PMC5505997; doi:10.1038/s41598-017-05287-z)
Supplement: Supplementary file 1 — supporting information [file 41598_2017_5287_MOESM1_ESM.pdf]

Flavonoid Enhances the Glyoxalase Pathway in Cerebellar Neurons to Retain its Functions

Joel Frandsen and Prabagaran Narayanasamy*

Department of Pathology and Microbiology, College of Medicine,

University of Nebraska Medical Center, Omaha, NE, 68198-5900

Corresponding author: p.narayanasamy@unmc.edu

Supporting information:

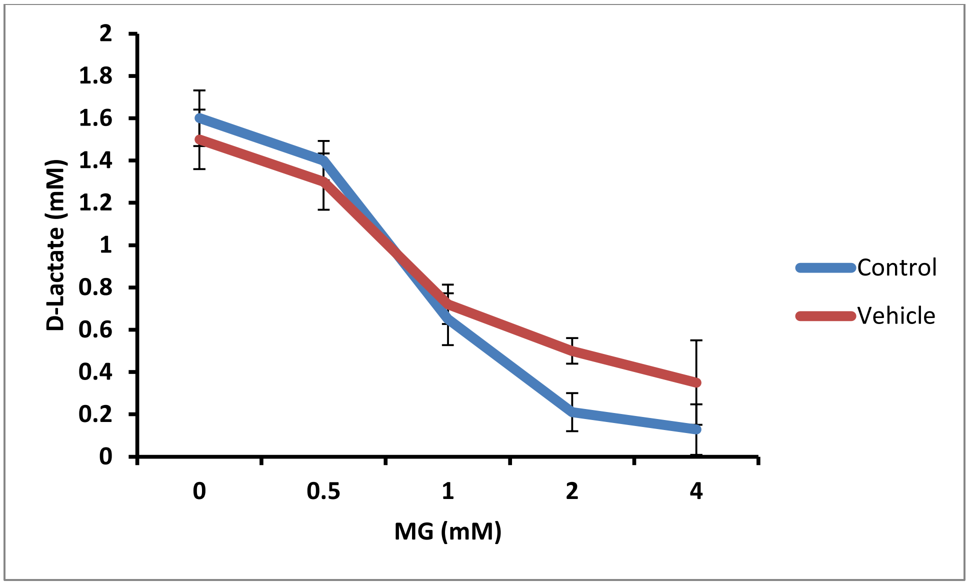

Figure S1: D-lactate assay determining experimental MG concentration. A D-lactate assay was performed with cerebellar neurons to determine the most efficient MG concentration for a model of aging and oxidative stress. Cerebellar neurons were treated for 24 hours with varying concentrations of MG (0-4 mM). The vehicle condition was treated with 0.01% DMSO. A MG concentration of 500 μM was determined to be the most efficient in treating cerebellar neuron cells.

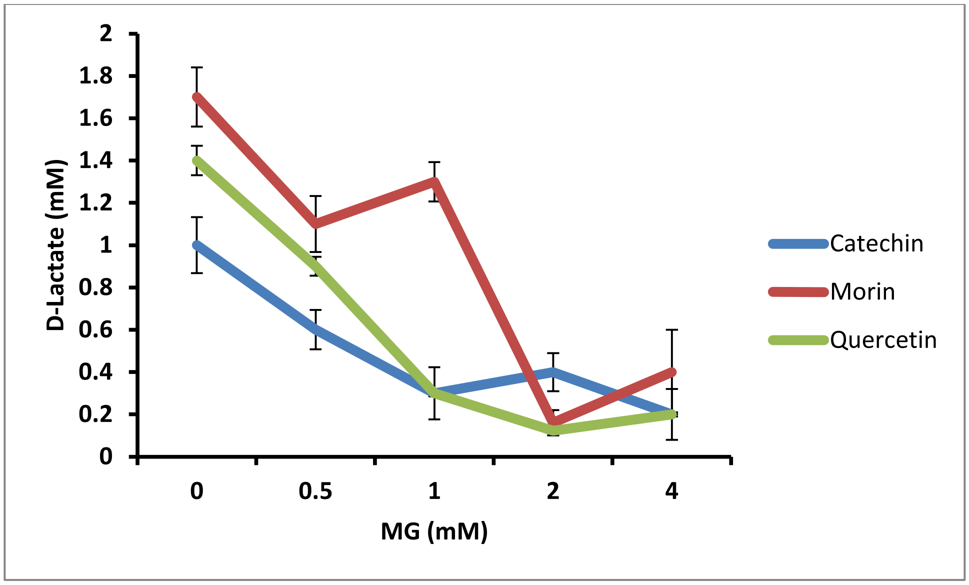

Figure S2: D-lactate assay determining experimental MG concentration. A D-lactate assay was performed with cerebellar neurons to determine the most efficient MG concentration for a model of aging and oxidative stress. Cerebellar neurons were treated for 24 hours with varying concentrations of MG (0-4 mM), and 10 μM of either catechin, morin, or quercetin.

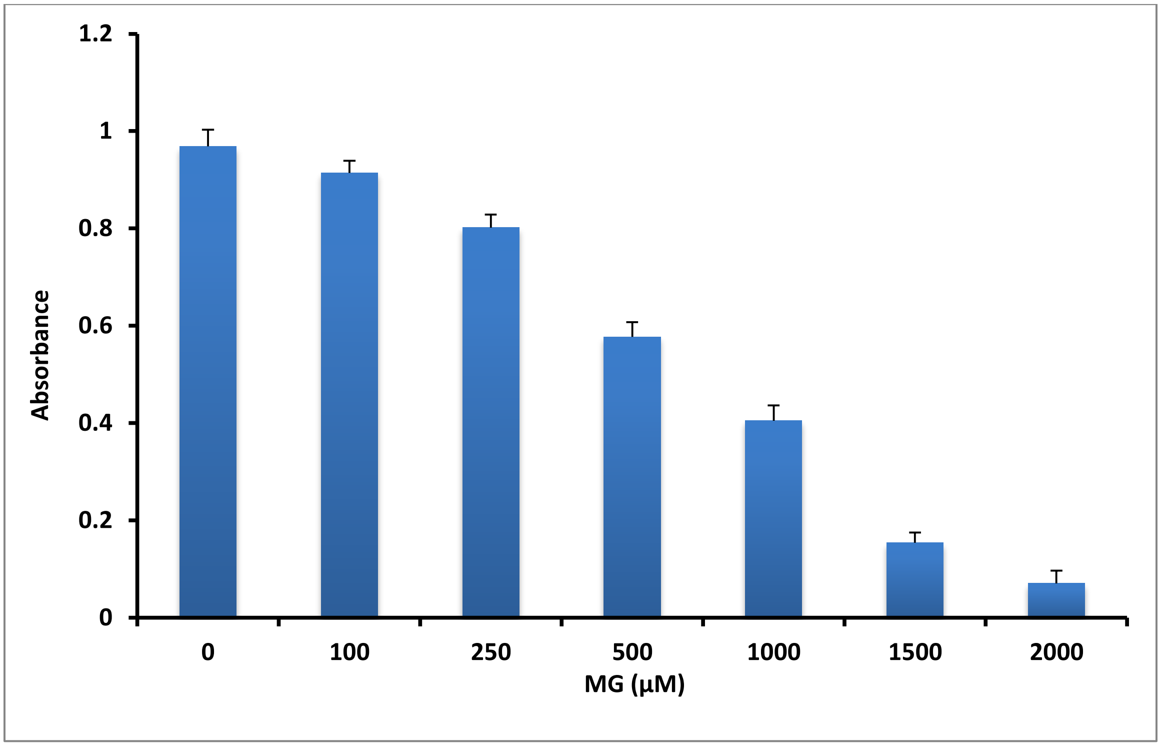

Figure S3: MTT assay for cerebellar neurons. Cerebellar neurons were grown in a 96 well plate at a density of 15,000 cells/well. Neurons were treated with varying doses of methylglyoxal for 24 hours. The media was collected and an MTT assay determining cell viability was measured absorbance at 570 nm.

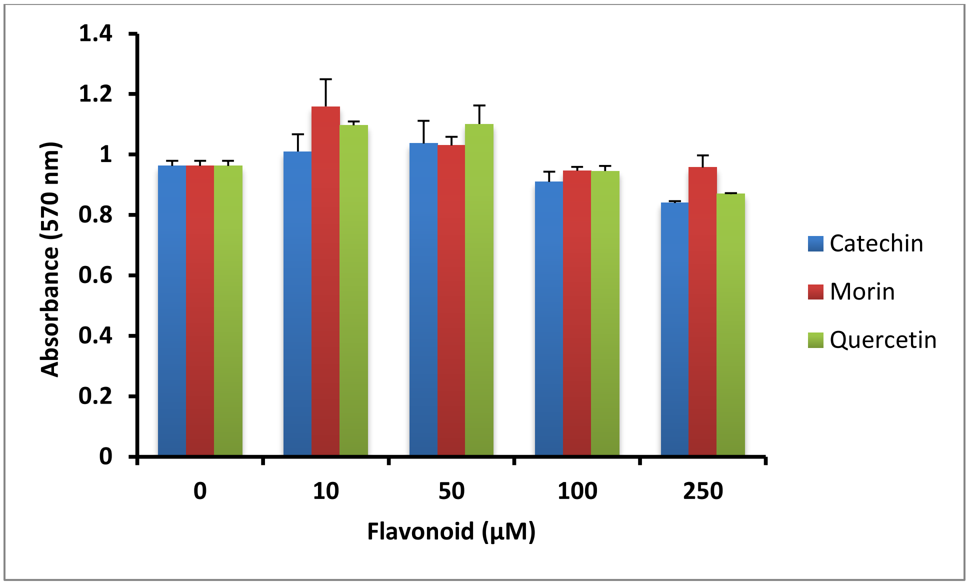

Figure S4: MTT assay for cerebellar neurons. Cerebellar neurons were grown in a 96 well plate at a density of 15,000 cells/well. Neurons were treated with varying doses of flavonoids (catechin, morin, and quercetin) for 24 hours. The media was collected and an MTT assay determining cell viability was measured absorbance at 570 nm.

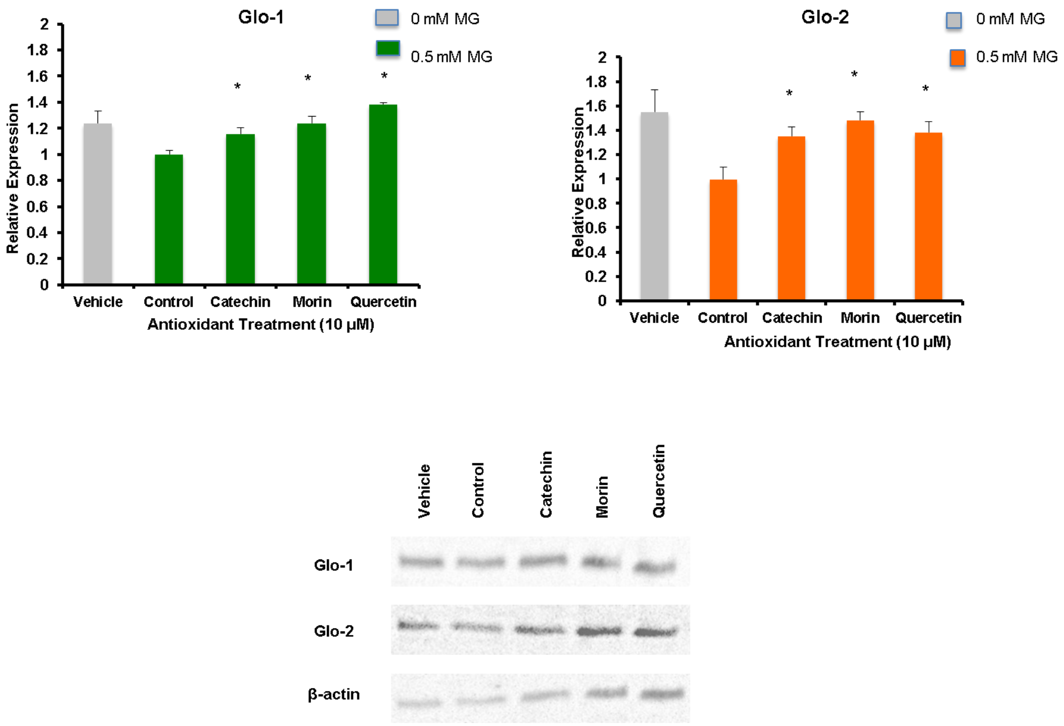

Figure S5: Glo-1 and Glo-2 protein expression in cerebellar neurons were assed via Western Blotting. Multiple experiments were performed, and representative bands are presented. Data was analyzed using a student's t-test (*p<0.05). A significant increase in Glo-1 and Glo-2 expression was found in the catechin, quercetin and morin treated cells, respectively.
